# Tracking with (Un)Certainty

**DOI:** 10.3390/jintelligence8010010

**Published:** 2020-03-03

**Authors:** Abe D. Hofman, Matthieu J. S. Brinkhuis, Maria Bolsinova, Jonathan Klaiber, Gunter Maris, Han L. J. van der Maas

**Affiliations:** 1 Department of Psychological Methods, University of Amsterdam, 1018 WS Amsterdam, The Netherlands; 2 Oefenweb, 1011 VL Amsterdam, The Netherlands; 3 Information and Computing Sciences, Utrecht University, 3584 CC Utrecht, The Netherlands; 4 ACTNext, Iowa City, IA 52243, USA

**Keywords:** computerized adaptive learning systems, student modelling, tracking, statistical inferences

## Abstract

One of the highest ambitions in educational technology is the move towards personalized learning. To this end, computerized adaptive learning (CAL) systems are developed. A popular method to track the development of student ability and item difficulty, in CAL systems, is the Elo Rating System (ERS). The ERS allows for dynamic model parameters by updating key parameters after every response. However, drawbacks of the ERS are that it does not provide standard errors and that it results in rating variance inflation. We identify three statistical issues responsible for both of these drawbacks. To solve these issues we introduce a new tracking system based on urns, where every person and item is represented by an urn filled with a combination of green and red marbles. Urns are updated, by an exchange of marbles after each response, such that the proportions of green marbles represent estimates of person ability or item difficulty. A main advantage of this approach is that the standard errors are known, hence the method allows for statistical inference, such as testing for learning effects. We highlight features of the Urnings algorithm and compare it to the popular ERS in a simulation study and in an empirical data example from a large-scale CAL application.

## 1. Introduction

One key ambition in educational technology is the move towards personalized learning. This development holds the promise of making tailor-made education available to everyone through online systems, allowing each learner to maximally realize their learning potential and improve both the learning process and learning outcomes. To this end, large-scale computer adaptive learning (CAL) systems are developed. These systems are designed to dynamically adjust the level or type of practice and instruction materials based on an individual learner’s performance. These systems should also provide diagnostic feedback on which skills and abilities the learner is deficient in, navigational support regarding which skills to work on, and reference to learning resources to improve these skills.

To measure the learners ability in CAL systems different learner models and estimation algorithms have been proposed. Different aims of CAL systems require different learner models. For example, in Bayesian Knowledge Tracing (BKT) skills are conceptualized as dichotomous variables that can be either in a learned stated or in an unlearned or forgotten state. In Item Response Theory (IRT) skills are conceptualized as continuous variables^1^; This conceptualization of skill is just one of many aspects that guides the choice of suitable learner models; see Pelánek et al. (2017) for an overview.

IRT includes a wide range of models of which the Rasch model (a logistic model) is the basic model. In the Rasch model the probability of a correct response is defined as follows:

(1) $$\mathrm{Pr}\left(X=1|\theta_{p},\beta_{i}\right)=\frac{\exp\left(\theta_{p}-\beta_{i}\right)}{1+\exp\left(\theta_{p}-\beta_{i}\right)},$$

where $\theta_{p}$ is the skill of person *p* and $\beta_{i}$ is the difficulty of item *i*. Commonly, the model parameters are estimated using off-line data, and assumed to be stationary. However in a CAL system a different approach is required. In CAL systems it is necessary to allow the model parameters, like ability, to change over time. This means that the model parameters need to be tracked while the data comes in. Different ‘online’ algorithms to estimate the key parameters in these systems have been proposed (Herbrich et al. 2006; Glickman 2001; Brinkhuis and Maris 2019a; Brinkhuis et al. 2015; Abbakumov et al. 2018). Ideally, model parameters are tracked when they are changing, yet show a known error distribution around their true value if they are stable (Brinkhuis and Maris 2019b).

The best known tracking system is the Elo Rating System (ERS) (Elo 1978). Klinkenberg et al. (2011) introduced an extended version of the ERS as a learner model for CAL systems. Since then, the ERS has been adopted in different learning systems (e.g., mathematics (Klinkenberg et al. 2011), touch typing (Van Den Bergh et al. 2015), Dutch language learning (de Bree et al. 2017) and geography facts (Papoušek et al. 2016)). The ERS is suited for CAL systems because it provides a simple and fast updating rule for parameter estimation. However, the ERS also has some drawbacks. In this paper we introduce a new algorithm that overcomes these drawbacks. We will highlight the statistical properties of the algorithm using a simulation study and a real data example from the Math Garden (Straatemeier 2014), a large CAL system for learning mathematics. Before we introduce the algorithm, we explain the ERS.

### 1.1. Elo Rating System

The ERS originates in chess, where players compete against other players, see for example Elo (1978) and Batchelder and Bershad (1979). Every game has an outcome (win, loss or draw) and after the match this observed outcome is compared to the expected outcome based on the differences between the ratings of both players before the game. This difference in outcomes is used to update ratings.

The ERS, specifically Elo’s current rating formula for continuous measurement, is introduced as follows (Elo 1978) [p. 25]:

(2) $$R_{n}=R_{o}+K\left(W-W_{e}\right)$$

$R_{n}$ is the new rating after the event.
$R_{o}$ is the pre-event rating.
*K* is the rating point value of a single game score.
*W* is the actual game score, each win counting 1, each draw 1/2.
$W_{e}$ is the expected game score based on $R_{o}$.

In a match between two players, each of the players has a rating $R_{o}$ before a match. The rating $R_{n}$ is increased if he or she wins and decreased in the case of a loss. The amount of increase or decrease in rating depends on the difference in ratings before the match. This difference determines the expected game outcome $W_{e}$ according to some measurement model, for example the logistic function in Equation (1). The ERS in Equation (2) shows that if a player performs according to expectation the update of the rating is small, and larger otherwise.

With some adaptations the ERS can be used in an educational measurement context. A large scale application of this idea can be found in Math Garden (Klinkenberg et al. 2011).

### 1.2. Math Garden

In Math Garden children play games in which they practice different mathematical or cognitive skills. In each game items are administered in an adaptive way using the ERS. Klinkenberg et al. (2011) introduced the ERS in an educational setting. To this end, the second player update is replaced with an item update following the idea that a player competes against an item in CAL systems:

$$\theta_{p(new)}=\theta_{p(old)}+K\left(S-E\left(S\right)\right)$$

$$\beta_{i(new)}=\beta_{i(old)}-K\left(S-E\left(S\right)\right).$$

where *S* is the observed score, and E(S) the expected scores of player *p* on item *i*, based on an extended Rasch model (Maris and Van der Maas 2012).

Since the introduction of Math Garden in 2012, a lot of schools have started to use the system for additional training. Currently, 714,000 users (both from family accounts or from one of the 2138 participating schools in the Netherlands) have made about 831 million responses, that are now collected at a rate of about one million a day.

**Figure 1:**
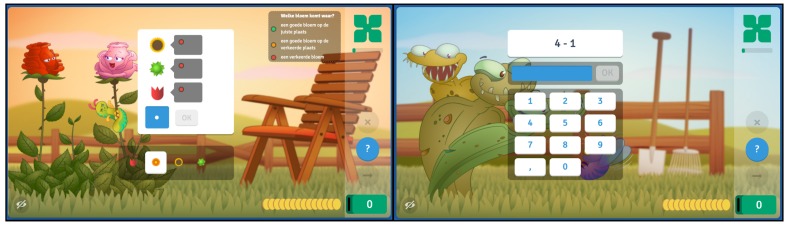
A screenshot of a single item in the Deductive Mastermind game (**left**) and in the Subtraction game (**right**). In the Deductive Mastermind game the coloured circles refer to either a correctly placed flower (green), a correct flower at the wrong location (orange) or a wrong flower (red). For this item the orange flower is the correct solution.

After logging in, a child lands on a page with different plants representing games for different mathematical and cognitive domains (e.g., addition, subtraction or a logical reasoning task). If a child starts a game a sequence of items is presented that are tailored to the ability of the child (see Figure 1 for an example item of the logical reasoning task (Mastermind) and an example item of the Subtraction game). A child can use the numpad to submit an answer or press the question-mark button to refrain from answering. The coins at the bottom, one disappearing each second, represent the scoring-rule that is used (Maris and Van der Maas 2012; Klinkenberg et al. 2011), and turn green after a correct response or red after an incorrect response. The interested reader can play the games using a free demo account; https://www.oefenweb.com/demo.

### 1.3. Research with Math Garden

The popularity of Math Garden provides researchers with an invaluable data set to study cognitive strategies and developmental patterns in learning. This research can be broadly categorized in three different lines. The first line is based on analyses of the rankings of persons and/or items following from the Elo measurement system. These analyses aim to understand differences in persons and/or items parameters. Examples of this research line are Klinkenberg et al. (2011), van der Ven et al. (2015, 2017), Gierasimczuk et al. (2013) and Jansen et al. (2014). For example, Klinkenberg et al. (2011) showed that the estimated person parameters of various arithmetic games in Math Garden correlate highly with paper and pencil tests. Additionally, the work of van der Ven et al. (2015, 2017) showed that item parameters match the effects predicted by different theoretical models about mathematics. Furthermore, Gierasimczuk et al. (2013) and van der Maas and Nyamsuren (2017) showed that the person and items parameters in both the Deductive Mastermind game and a Number Series game can be explained by substantive models developed for these cognitive tasks.

A second research line is aimed at understanding the cognitive strategies used by players in Math Garden. To this end, the ‘raw’ responses (accuracy and response times) to items of a subset of children who played a certain game on a regular basis are analyzed with an extended latent variable model (e.g., Hofman et al. 2018a). For example, Hofman et al. (2015) reanalysed data from the balance-scale task (Siegler 1976) and compared a rule-based model and an information-integration model.

A third line aims at investigating developmental processes using longitudinal data (e.g., Van Den Bergh et al. 2015). Hofman et al. (2018b) investigated the mutual developmental links between different math skills as predicted by the mutualism model of intelligence (van der Maas et al. 2006).

### 1.4. Challenges in Elo Rating Systems

One of the drawbacks of the ERS is that ratings have no known error distribution when person ability and item difficulty parameters are stationary (Brinkhuis and Maris 2008; Brinkhuis and Maris 2009; Brinkhuis and Maris 2019b). When standard errors of the ratings are unknown, statistical inference on ratings is not possible. In the context of CAL, statistical inference is desirable since it allows, for example, to test for the growth in ability, test whether one differs from a reference group, and test whether item difficulties change after an intervention.

A second problem is rating variance inflation, which we illustrate both by simulation and in a real data example. We simulated 4000 games of 10 sessions, based on the Rasch model, of 200 players to 50 items. After each response we updated the parameters with the ERS and based on these parameters the next item is selected using an item selection procedures that results in an average of about 50% correct responses. The *K*-factor is fixed at 0.25. For both items and persons the real values are standard normally distributed and the initial values are all set to zero.

**Figure 2:**
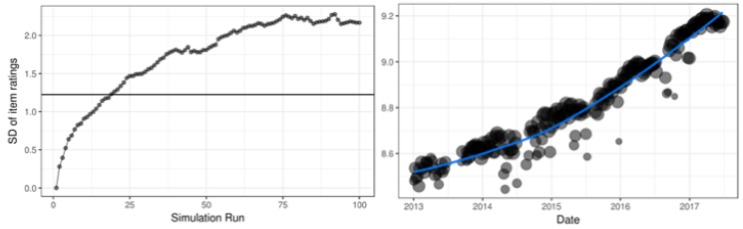
Rating variance inflation in a simulated (**left**) and real data example (**right**) using the Elo Rating System (ERS).

The left panel of Figure 2 shows the problem of rating variance inflation in the ERS simulation. Since all starting values are zero it takes some runs until the system converges. After about 20% of the simulated games, the observed variance crosses the horizontal line indicating the true variance. However, in the remaining runs the variance keeps increasing. This indicates that the scale at which the system operates is not fixed over time. It should be noted that the magnitude of the effect is determined by a lot of factors: For example, the *K*-factor, the numbers of items and persons in the system and the item selection function. However, in all situations where items are adapted to the ability of players some inflation of rating variance occurs. The right panel of Figure 2 shows a comparable increase in the standard deviation of the item ratings of the subtraction game for every week between January 2013 and September 2017.

While this inflation biases the ratings, the ranking of the players (items) remains intact. Hence, the adaptivity of the CAL is not compromised. However, due to this rating variance inflation comparisons between Elo ratings at different time points are troublesome. Drift of the rating scale, and change of the rating pool is known to be a problem.^2^

### 1.5. Alternatives to Elo Rating Systems

There are two popular alternatives to Elo Rating Systems we like to discuss, namely Glicko (Glickman 2001) and TrueSkill (Herbrich et al. 2006; Minka et al. 2018). Both these rating systems allow for tracking player abilities with some measure of uncertainty about them. Glicko is an updating algorithm for a Gaussian state-space model, and TrueSkill is based on Gaussian density filtering. Both systems make specific distributional assumptions of normality of skills and of drift of skills between time steps. These Gaussian distributions allow for efficient approximations of posterior densities of skills, even on very large data sets (Minka et al. 2018).

### 1.6. Three Problems in Rating Systems

We can identify three problems in rating systems, such as the ones mentioned before, that seem to not be fully recognized in the literature.

First, and already mentioned, there is the issue that the invariant distribution of ratings is generally unknown. Most rating systems (such as Elo (1978) and Glickman (2001)) generate a Markov chain, but the invariant distribution of these Markov chains are unknown. Second, and more importantly, in the invariant distribution for a pair of players the marginal distribution for one player depends on the skill of the opponent. Hence the invariant distribution of the Markov chain changes as players compete against different opponents. Third, the main use of rating systems is to pair players of about equal strength, or players to items (match making). Suppose we have a collection of transition kernels, each with the same invariant distribution. If we choose a transition kernel from the collection based on the current state of the Markov chain, the invariant distribution is no longer retained. In the Appendix A we demonstrate the importance of correcting for this effect of adaptive match making. In the next section, we provide an alternative for the ERS that resolves these three issues.

### 1.7. Outline

In the following, we introduce a new algorithm that is suited to track both player ability and item difficulty parameters. Importantly, this algorithm also provides (1) standard errors indicating the (un)certainty in these parameters, and (2) a way of correcting the parameters to prevent rating variance inflation. First, we describe the algorithm. Second, we highlight some of the new properties using simulations. Third, we will apply the Urnings algorithm to data collected with Math Garden and highlight some of the additional inferences that the standard errors provided by Urnings allow us to test.

## 2. Methods

### 2.1. The Urnings Algorithm

We start by restating the Rasch model as a game of chance in which both the player and the item are represented by an urn. These urns are thought to have an infinite number of green and red marbles. The proportion of green marbles in these urns equals the (inverse logit of the) player ability and item difficulty. The game proceeds as follows. Two marbles are drawn -one from the urn of the player and one from the urn of the item- until they are of different colors. The player wins (i.e., a correct response is given) when a green marble was drawn from the player’s urn and a red marble was drawn from the item’s urn, and the player loses (i.e., an incorrect response is given) if a red marble was drawn from the player’s urn and a green one from the item’s urn. See Algorithm 1 for an algorithmic representation of this game of chance.
**Algorithm 1:** Game of Chance **repeat**  $Y_{p}∼Bernoulli\left(\pi_{p}\right)$  $Y_{i}∼Bernoulli\left(\pi_{i}\right)$ **until**
$Y_{p}\neq Y_{i}$ **return**
$X_{pi}=Y_{p}$

Where $X_{pi}$ is the outcome of the game (1 for a correct response, 0 for an incorrect response), $\pi_{p}=\frac{\exp(\theta_{p})}{1+\exp(\theta_{p})}$ and $\pi_{i}=\frac{\exp(\beta_{i})}{1+\exp(\beta_{i}))}$ are the inverse logit transformed ability of player *p* and difficulty of item *i*, respectively. It can be shown that the probability of a correct response in a game of chance like this is the same as in the Rasch model (compare Equation (4) with Equation (1). As the outcome ($y_{p}$, $y_{i}$) occurs with probability:

(3) $$\mathrm{Pr}\left(Y_{p}=y_{p},Y_{i}=y_{i}\right)=\pi_{p}^{y_{p}}{\left(1-\pi_{p}\right)}^{\left(1-y_{p}\right)}\pi_{i}^{y_{i}}{\left(1-\pi_{i}\right)}^{\left(1-y_{i}\right)},$$

we obtain by conditioning on $Y_{p}$ not being equal to $Y_{i}$ that:

(4) $$\begin{array}{cc} \mathrm{Pr}\left(Y_{p}=1,Y_{i}=0|Y_{p}\neq Y_{i}\right)=\mathrm{Pr}\left(X_{pi}=1\right) & =\frac{\pi_{p}\left(1-\pi_{i}\right)}{\pi_{p}\left(1-\pi_{i}\right)+\pi_{i}\left(1-\pi_{p}\right)}=\frac{\frac{\pi_{p}}{1-\pi_{p}}\frac{1-\pi_{i}}{\pi_{i}}}{\frac{\pi_{p}}{1-\pi_{p}}\frac{1-\pi_{i}}{\pi_{i}}+1}\\ & =\frac{\exp(\theta_{p}-\beta_{i})}{\exp(\theta_{p}-\beta_{i})+1}. \end{array}$$

The proportion of green marbles in the urn might change over time because players may become more proficient. In the rating system we want to mimic the game of chance that takes place in reality and track the proportions of green marbles over time. To do that, we will represent the rating of the player/item with finite urns consisting of *n* marbles with *u* green marbles and design a tracking system in such a way that the invariant distribution of *u* would be a binomial distribution with parameters *n* and π. Then we can track the ability of each player *p* and the difficulty of each item *i* by tracking $\frac{u_{p}}{n_{p}}$ and $\frac{u_{i}}{n_{i}}$, respectively. See Algorithm 2 for an algorithmic representation of the same game of chance, providing an expected match outcome based on the introduced parameters.
**Algorithm 2:** Game of Chance with Urnings **repeat**  $Y_{p}^{*}∼Bernoulli\left(u_{p}/n_{p}\right)$  $Y_{i}^{*}∼Bernoulli\left(u_{i}/n_{i}\right)$ **until**
$Y_{p}^{*}\neq Y_{i}^{*}$ **return**
$X_{pi}^{*}=Y_{p}^{*}$

We can start the system with either a random composition of marbles in the urns of the players and the items, or with half of the marbles being green and half red in all urns. To update the urns we remove the marbles from the last draw (expected match outcome) and replace them with the outcome of the actual game: If the player solved the item correctly a green marble is added to the urn of the player and a red marble is added to the urn of the item, and vice verse if the actual response was incorrect. The proposed update can be represented as follows:

$$\begin{array}{cc} u_{p}^{*} & =u_{p}+X_{pi}-X_{pi}^{*}\\ u_{i}^{*} & =u_{i}+\left(1-X_{pi}\right)+\left(1-X_{pi}^{*}\right) \end{array}$$

However, this update is only a proposal which might or might not be accepted by the system with a specific probability specified to ensure that the constructed Markov chains converge to their appropriate invariant distributions (i.e., the Metropolis–Hastings algorithm is employed (Chib and Greenberg 1995; Brinkhuis and Maris 2010)). The Metropolis–Hastings step is needed to make sure that the invariant distributions of $u_{p}$ and $u_{i}$ are independent of each other. In the Metropolis–Hastings step the the proposed values are accepted with probability:

(5) $$\mathrm{Pr}\left(\left[u_{p},u_{i}\right]\to \left[u_{p}^{*},u_{i}^{*}\right]\right)=\min\left(1,\frac{u_{p}\left(n_{i}-u_{i}\right)+\left(n_{p}-u_{p}\right)u_{i}}{u_{p}^{*}\left(n_{i}-u_{i}^{*}\right)+\left(n_{p}-u_{p}^{*}\right)u_{i}^{*}}\right).$$

Additionally, if the player and the item are matched to each other in an adaptive way based on their current ratings in the system (which is often the case in CAL systems), then an extra term is added to the acceptance probability such that we take into account that after the proposed update the probability of player *p* being matched to item *i* changes:

(6) $$\mathrm{Pr}\left(\left[u_{p},u_{i}\right]\to \left[u_{p}^{*},u_{i}^{*}\right]\right)=\min\left(1,\frac{u_{p}\left(n_{i}-u_{i}\right)+\left(n_{p}-u_{p}\right)u_{i}}{u_{p}^{*}\left(n_{i}-u_{i}^{*}\right)+\left(n_{p}-u_{p}^{*}\right)u_{i}^{*}}\frac{M_{pi}\left(u^{*}\right)}{M_{pi}\left(u\right)}\right),$$

where $M_{pi}\left(u\right)$ is the probability of item *i* being matched to player *p* given the ratings of the player and all the items in the system. This addition is an important feature of the Urnings algorithm. It guarantees that match making resulting from the adaptive item selection does not lead to rating variance inflation (see also Appendix A or the more detailed description in Maris et al. (2020)). We note that alternative rating algorithms that implement some form of standard errors, such as (Glickman 2001; Herbrich et al. 2006; Minka et al. 2018) do not correct for match making by adaptive opponent or item selection.

Note, that the size of the urns can be different for players and items. The size of the urn relates to *K* parameter in the ERS. With larger *n* there is less noise in the ratings and the correlation between the true values and the ratings will, in long term, be higher. However, with large *n* it will take more item responses to track changes in ability/difficulty if they occur.

With the algorithm described above, the total number of green marbles in the system remains constant for all players and all items. However, items might be added to or removed from the system, and players might be entering and leaving the system over time. To keep the scale of ability/difficulty constant across time, one can define a subset of core items in the system and keep the number of green marbles constant for that subset. If a green marble is added to (and the red marble is removed from) the urn of one of the items in the core subset, then a green marble is removed from the urn of another randomly selected item in the core subset and a red marble is added to it. The opposite happens if a red marble is added to the urn of one of the items in the core subset. By keeping the number of green marbles for the core subset constant, the abilities of the players and the difficulties of the items can always be interpreted in the same way in relation to the items of the core subset.

### 2.2. Simulation Setup

We performed a simulation study to test whether the ratings in the Urnings system track the theoretical values based on the implied binomial distributions depending on the urn size and true value $\pi_{p}$ or $\pi_{i}$. To this end we simulated responses of 500 players to 100 items, where $\pi_{p}∼N\left(0,1\right)$ and $\pi_{i}∼N\left(0,1\right)$. We simulated 1,000,000 sessions where a random player makes a set of 10 items. These items were adaptively selected based on the current ratings, such that $\mathrm{Pr}(X=1\;|\;u_{p},u_{i})\approx 0.5$. The probability of selecting an item was proportional to the normal density with mean equal to the difference between the logits of $u_{p}/n_{p}$ and $u_{i}/n_{i}$ and a SD of 1.^3^

The urn sizes for persons were set to 60 and for items to 200, reflecting the higher precision for the more frequently updated item parameters compared to the person parameters. As starting values all urns were filled with 50% of green and 50% of red marbles. For all items and all persons, except one person, the true values of their difficulties and abilities were stable across the simulation. For one person a change in their ability was included to demonstrate how the Urnings system adapts to changing ability.

## 3. Results

### 3.1. Simulation Results

**Figure 3:**
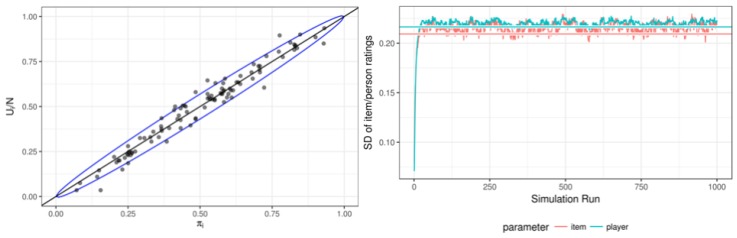
The left panel shows the true $\pi_{i}$ versus estimated $u_{i}/n_{i}$ item ratings, and the 95% confidence interval (CI) implied by the urn size. The right panel shows the SD of the item and person ratings throughout the simulation. The horizontal lines reflect the SD based on the samples from the true values.

The left panel of Figure 3 shows the true versus estimated item parameters, with a 95% confidence interval (CI) as implied by the urn sizes. The ratings highly correlate with the true values (ρ=0.981 for the item parameters; ρ=0.962 for the person parameters). The magnitude of the correlation relates to the urn sizes, as is reflected by the higher correlation for items induced by the larger urn sizes for items compared to persons. Furthermore, 95.2% of the person ratings reside inside the CI indicating proper coverage of the CI. For the items, 92% of the ratings reside inside the CI.^4^

**Figure 4:**
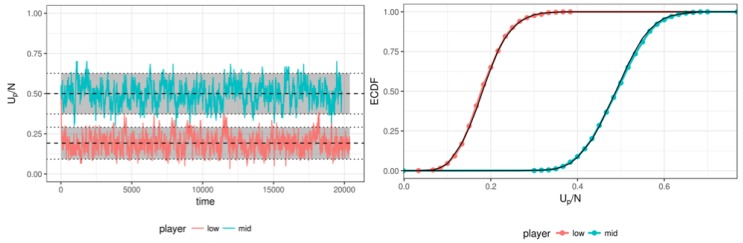
The left panel depict the estimates of two players (one with a low and one with medium true value throughout the simulation, with the grey bars indicating the 95% CI. The right panel shows close correspondence of the cumulative density function based on the estimates (coloured dots) and based on the true values (black line).

Additionally, the right panel of Figure 3 shows the standard deviation of the ratings during the simulation, which matches the expected standard deviation of the true values.^5^ Note the stability of the estimated standard deviation compared to the left panel of Figure 2. We also compared the ratings over time (in a stationary state) to the expected distributions for two random persons, see Figure 4. The left panel shows that the estimated ratings follow the true values (dashed lines) and approximately follow the expected distribution, as depicted by the cumulative density function of the estimates in the right panel.

**Figure 5:**
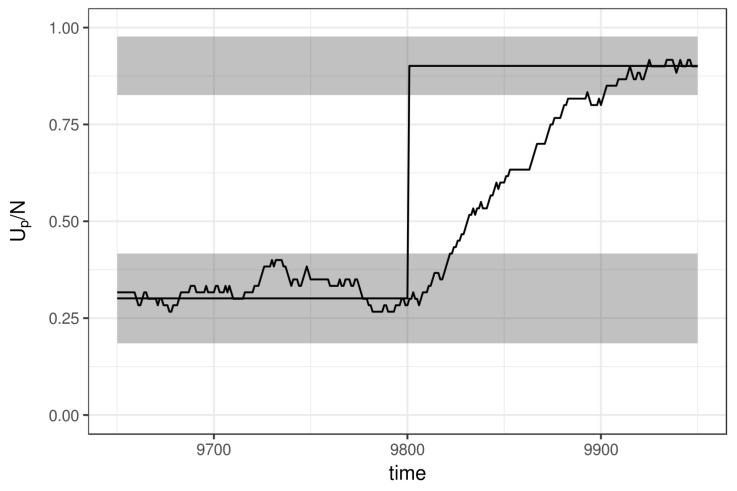
The tracked rating follows the true value of a simulated player that showed a jump in ability at iteration 9800.

To show that the ratings adapt to the new true state (learning), Figure 5 depicts the ratings ($u_{p}/n_{p}$) of a simulated player that at iteration 9800 suddenly changed its true ability. The tracker nicely follows this jump and after nine sessions (93 responses) the tracker falls within the 95% CI of the new true value. Note that the speed of convergence to a new state is, other than the size of the jump, solely dependent on the urn size.

### 3.2. Real Data Example: Math Garden

#### 3.2.1. Description of the Data

To run the algorithm, we selected data of two different games from the Math Garden system. First, in a deductive version of the Mastermind game—a logical reasoning task—children need to use different cues to solve problems with a single correct solution. See Gierasimczuk et al. (2013) for a detailed description of the game. Second, in the Subtraction game children practice different subtraction items ranging from easy (e.g., 5–2) to more difficult items (e.g., 97–28).

For the Mastermind and Subtraction game data were respectively collected between 2015-01-01 and 2019-06-30 and between 2013-01-01 and 2017-06-30. For both games only players who provided at least 270 responses were selected. For the Mastermind games this resulted in data from 8616 players with 3,556,884 responses to 725 different items. For the Subtraction game data of 4310 players were selected resulting in a total of 1,784,457 responses to 508 items.

Furthermore, for both games we only selected responses of children who played at the hard difficulty level (Jansen et al. 2016). The question-mark response was treated as an incorrect response. The Urnings algorithm was initialized with equal numbers of red and green marbles for all item and player urns. As urn sizes we choose 80 for the items and 30 for the players.^6^ For the results we used a burn-in of three times the urn size.

Note that in the empirical example we cannot correct for adaptive item selection, because the system did not apply the Urnings algorithm but the ERS.

#### 3.2.2. Results

**Figure 6:**
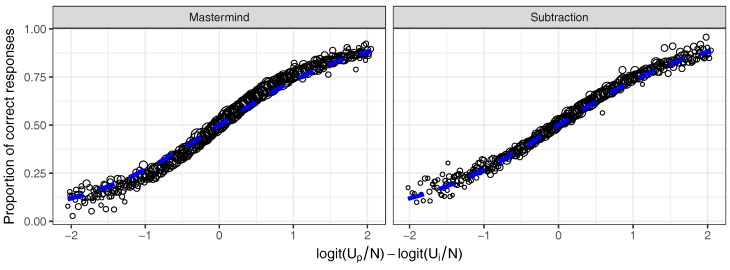
A visualisation of model fit for both analysed games, by comparing observed (black dots) and expected (blue line) probabilities of a correct response for each of the binned differences between the logits of $u_{p}/n_{p}$ and $u_{i}/n_{i}$.

First, Figure 6 shows that the ratings obtained with the Urnings algorithm predict the future responses in the system rather well. Figure 6 shows the average probability of the observed correct responses for different equally spaced bins based on the differences in the logits of $u_{p}/n_{p}$ and $u_{i}/n_{i}$. These averages are, for both analysed game, very similar to the expected probabilities implied by the Rasch model (blue line).

**Figure 7:**
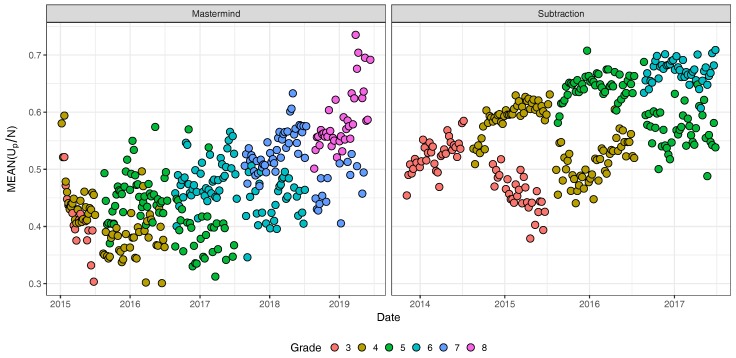
The average rating of players between 2015 and 2019 (Mastermind) and 2014 and 2017 (Subtraction) of children born in 2007.

Second, to illustrate learning in the Math Garden system we follow a cohort of children born in 2007. Data are aggregated by week. Only the average scores are included of weeks on which at least 25 different players have played the game. The left panel of Figure 7 shows that when children become older an increase is present the average estimated ability scores in the Deductive Mastermind game. However, also a big variation is present in the scores between different weeks. For the subtraction game, as expected, the average ability rating increased over time (right panel of Figure 7). The lower cluster of dots represent children that lack a year behind the expected grade. A comparison of the two panels highlights the expected difference between the average growth in ability in a scholastic game versus a non-scholastic game. Since children practise subtraction in school, large grade differences are expected and found in the data, whereas for the Mastermind game an increase over time is present that large grade effects are missing.

**Figure 8:**
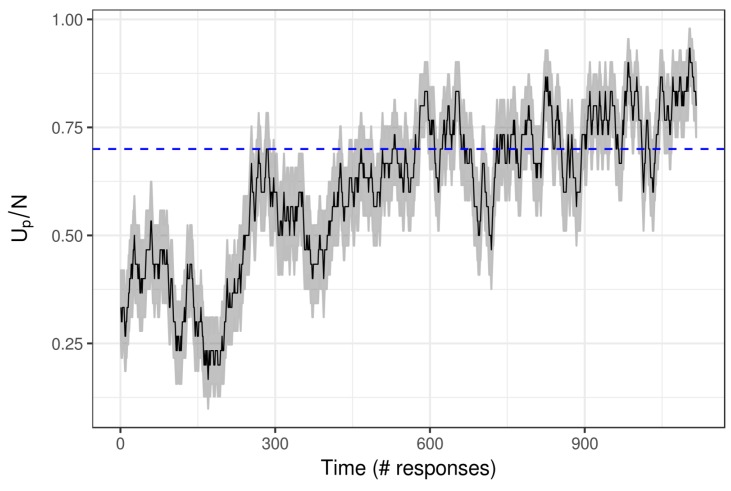
The rating development (and the CI in grey) of a player. The horizontal line at Up/N=0.7 indicates an (arbitrary) reference point that could indicate a sufficient ability in the subtraction domain.

Finally, we analyze the growth of a single player. Figure 8 shows the rating of a player during the 1116 responses (s)he provided. As indicated by the CI, in the first set of about 300 responses the ability of this player grows towards the reference point of $u_{p}/n_{p}=0.8$. From the 300th response on-wards, the rating first declines and then fluctuates around 0.7 (as indicated by the CI). Towards the end of this times series, the rating indicates that this player outperform the reference point. The rating corresponds to item ratings of item such as 26–19, 42–23, and 50–33.

## 4. Discussion

In this paper we presented a new way of tracking parameters in CAL systems. In current CAL systems the popular ERS provides an elegant way of estimating person and item parameters on the fly, that is while data come in. However, we showed that the ERS also has drawbacks. Current ratings systems suffer from three problems, namely a lack of an invariant distribution, opponent dependence, and matchmaking dependence.

We proposed a new Urnings algorithm to resolve these three problems. This algorithm provides known distributions for all model parameters, and corrects for both opponent dependence and adaptive match making by using Metropolis–Hastings. In doing so, we obtained a rating system with known error distributions and that tracks rating changes when they occur. Importantly, in comparison to Glicko and TrueSkill, this is achieved without specific distributional assumptions of normality of skills and of drift of skills between time steps.

Using both data simulations and a real data example we highlighted different features of the Urnings algorithm. Since the real data were not collected with an online implementation of the Urnings algorithm we could not correct for the adaptive item selection function. However, the simulated example shows that if the Urnings algorithm is fully implemented it also solves the rating variance inflation problem. This would allow the study of changes in the variance of ratings over time required, for instance, to test for the Matthew effect (Savi et al. 2018).

A key property of the Urnings rating system is that measurement accuracy is a design parameter. By setting the urn sizes we determine measurement accuracy. Given the urn size, the 95% CI interval is known, as long as the ratings are stationary (see Figure 3). In the current study the urn sizes are arbitrarily set and fixed over time. In practice, we probably would like to adapt the urn size. Large urns are good for precision in measurement but they are slow in tracking changes in true ratings. This bias-variance trade-off should be dealt with in each CAL system. In the ERS this is accomplished by adapting the *K*-factor. In the Urnings algorithm we could let urns sizes grow if observations come in frequently and decrease urn size when players play less. The cold-start problem (Park et al. 2018) could be partially solved by relating initial ratings for new players and items to different user or item characteristics.

We have used a simple Rasch model for the probability of a correct response to the item. The Urnings algorithm can be extended to allow for polytomous items, multidimensional abilities, varying item weights for the measurement of ability, and inclusion of response times. As long as the observed and expected outcome can be formulated as a simple game of chance based on infinite and finite urns of marbles, the appropriate extension of the Urnings algorithm can be formulated to track the item difficulties and player abilities.

To conclude, since CAL systems are becoming more widely adopted in education, the inferences made about individual children based on the reliable learning analytics provided by these systems should be a crucial part of designing the system (Brinkhuis et al. 2018; Hofman et al. 2018c). Based on these inferences tailored instructions could be provided, either automated within the system or by teachers in a classroom setting.

## Figures and Tables

**Figure A1:**
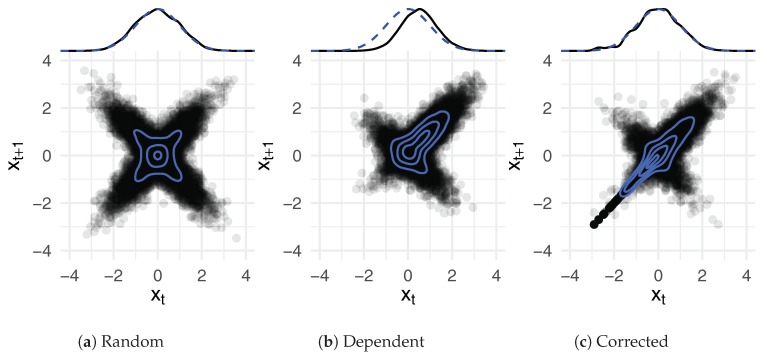
Joint distribution of the current and the new states of the Markov chain and the invariant distribution of the chain (black curve on top) compared to the standard normal distribution (blue dashed curve). where (**a**) the transition kernel is selected randomly, (**b**) the transition kernel is selected dependent on the current state, and (**c**) the transition kernel depends on the current state, but this dependence is corrected for in a Metropolis–Hastings step.

## Footnotes

1. see Deonovic et al. (2018) for a description of the relation between IRT and BKT.

2. In international chess competitions, this is also recognized, the FIDE handbook describes how rating drift should be monitored in article 10, https://www.fide.com/fide/handbook.html?id=197&view=article.

3. The interested reader can find the simulation code in the following OSF project: https://osf.io/8wgvb/.

4. This is lower than for the persons, but not inconsistent with the 95% confidence interval since there are only 100 items.

5. In comparing observed and expected rating distributions, the proper error distribution is added to the expected ratings, see for example (Brinkhuis 2014).

6. The interested reader can find the code to estimate the Urnings algorithm in this OSF project: https://osf.io/8wgvb/ and access to the data can be acquired by contacting the first author.

## Abbreviations

The following abbreviations are used in this manuscript:

ERS	Elo Rating System
CAL	Computer Adaptive Learning
BKT	Bayesian Knowledge Tracing
IRT	Item Response Theory

## Appendix A. Illustration of the MH-Step

In the actual use of rating systems, the current ratings of players are often used to determine who will play against whom next. This is not an innocent activity though, as it has the potential to effect the invariant distribution. This is a general phenomenon, and is best illustrated in the general case.

To illustrate the problem, let $p_{i}\left(x^{*}|x\right)$ be a collection of transition kernels each of which has the same invariant distribution π(x). If $f_{i}\left(x\right)$ denotes the frequency with which transition kernel *i* is selected when the current state is *x*, then even though

(A1) $$\begin{array}{cc} \forall & i:\pi \left(x^{*}\right)=\int_{R}p_{i}\left(x^{*}|x\right)\pi \left(x\right)dx \end{array}$$

we find that when kernels are selected depending on the current state then

(A2) $$\phi \left(x^{*}\right)=\sum_{i}\phi_{i}\left(x^{*}\right)=\sum_{i}\int_{R}p_{i}\left(x^{*}|x\right)f_{i}\left(x\right)\pi \left(x\right)dx$$

which is not necessarily our invariant distribution (ϕ≠π).

We present an example to illustrate this not a very intuitive property. Let us consider a Markov chain for which we want a standard normal as its invariant distribution. A conditional of the multivariate normal with zero means, unit variance and any correlation given the current value can serve as a proper transition kernel for our invariant distribution. The value of the correlation can also change across iterations, for example take values of 0.9 and −0.9 with the probability of 1/2. Figure A1a shows the joint distribution of $x_{t}$ and $x_{t+1}$: It is clear that each of the kernels is used in half of the iterations and the resulting marginal distribution is standard normal. However, if the value of the correlation depends on $x_{t}$, then the invariant distribution will no longer be standard normal. For example, if the transition kernel $x_{t+1}∼N\left(0.9x_{t},1-0.9^{2}\right)$ is selected with the probability ${\left(1+\exp\left(-2x_{t}\right)\right)}^{-1}$, then the part of the joint distribution in the lower left quadrant is missing and the marginal distribution is not standard normal (see Figure A1b). Metropolis–Hastings provides the correction (see Figure A1c).
